# Folliculosebaceous Cystic Hamartoma with Spindle Cell Lipomatous and Neural Components

**DOI:** 10.3390/dermatopathology10030027

**Published:** 2023-06-25

**Authors:** Carmelo Urso, Marina Yarygina

**Affiliations:** 1 Dermatopathology Study Center of Florence, I-50129 Florence, Italy; 2 Synlab Med, I-50041 Calenzano, Italy; 3 Synlab Italia, I-25014 Castenedolo, Italy; marina.yarygina@synlab.it

**Keywords:** folliculosebaceous cystic hamartoma, spindle cell lipoma, nerve, follicular tumors, hamartomas

## Abstract

Folliculosebaceous cystic hamartoma is a cutaneous malformation composed of a cystic folliculosebaceous structure associated with mesenchymal elements, generally consisting of fibrous stroma, adipocytes and small vascular channels. We report the case of a 55-year-old female patient with a cutaneous nodule of the right nasal wing. Microscopically, the lesion showed a dilated hair follicle with multiple sebaceous glands, surrounded by a mesenchymal component composed of fibromyxoid stroma, spindle cells, mature-appearing adipocytes and collagen bundles, resembling spindle cell lipoma, associated with an additional neural component, consisting of small nerve bundles. In folliculosebaceous cystic hamartoma, the association of spindle cell lipomatous and neural components has not previously reported.

## 1. Introduction

Folliculosebaceous cystic hamartoma is a cutaneous lesion, originally described in 1991 by Kimura et al., who described five cases of a distinct pilosebaceous malformation, proposing a set of specific diagnostic criteria [1]. In their paper, the authors used the term “hamartoma”, defined as “a jumble” of indigenous tissues, to indicate a malformative rather than a true neoplastic lesion, and the adjective “cystic” to signify “like a cyst but not a true cyst” [1]. Although infrequent, this lesion is not exceedingly rare, as in the past three decades more than 240 cases have been described [2]. More than 80% of these cases have been reported by Asian authors [2,3], including the largest series, accounting for 153 lesions, published by Ansai et al. in 2010 [3].

Folliculosebaceous cystic hamartoma is, probably, an underdiagnosed lesion, because of the lack of specific clinical features [2,4]. Clinically, it usually appears as a slow growing, skin colored, cutaneous papule or nodule, sessile or pedunculated. Some lesions may appear as red or dome-shaped nodules, sometimes showing superficial teleangiectasias or a warty appearance [2,3,4,5]. No statistically significant predominant sex incidence has been found [2,3,5]. In the largest published series, the age of patients ranged between 15 and 88 years old (mean 54.3 ± 15.4 years old) [3]. Typically, the lesion measures 2 cm or less and is mainly located on the head, most commonly on the face, often around the nose [3,5]; the most frequently involved sites are (in decreasing order): nasal/paranasal region, scalp, auricolar/periauricolar region, cheeks, forehead and mandible. Other possible less frequently involved anatomic locations include back, nipple, axilla and shoulder. A giant variant, measuring more then 2 cm, may occur in other anatomic districts, including back, vulva, scrotum, neck, upper extremities and the sacral area [2,3,5]. Duration of tumors may vary greatly, from 6 months to 20 years (average 7 years) [3]. In most of cases, a solitary lesion is observed, but multiple lesions may also occur [2]. They are generally asymptomatic and, rarely, have mild subjective symptoms, including a feeling of discomfort, pruritus and mild pressure pain, which have been recorded [2]. Clinical diagnoses include a large number of skin lesions, comprising an epidermal cyst, a melanocytic nevus, fibrous papule of the nose, soft fibroma, adnexal tumor, sebaceous hyperplasia, basal cell carcinoma, neurofibroma and benign soft tissue neoplasm [3,5,6]. Associations with nevus lipomatosus superficialis, Miescher-type melanocytic nevi, rosacea or port-wine stain have rarely been reported [5].

Histopathologically, folliculosebaceous cystic hamartoma appears as a complex lesion composed of epithelial, follicular and sebaceous, and mesenchymal tissues. The tumor shows a cystically dilated hair follicle with multiple radiating sebaceous glands, associated with a mesenchymal component, generally consisting of abundant fibrous stroma, small venules and a variable proportion of mature adipose tissue [1,2,3,4,5,6,7].

We report a case of folliculosebaceous cystic hamartoma showing the association of two additional rare mesenchymal features: a spindle cell lipomatous component, mimicking spindle cell lipoma, and a neural component, consisting of short nerve bundles embedded in the lesional stroma.

## 2. Report of a Case

A 55-year-old female patient presented a 10 × 9 mm non-pigmented, slowly enlarging, cutaneous nodule of the right wing of the nose. The lesion, previously treated by diathermo-coagulation, was removed by simple excision, with a provisional clinical diagnosis of intradermal nevus or fibrous papule. The patient has had no complications or recurrence. The specimen, measuring 12 × 9 × 7 mm, was fixed in 10% buffered formalin and entirely embedded in paraffin. The tissue sections were stained with hematoxylin-eosin for histologic examination; some sections were histochemically stained with Alcian blue. Additional tissue sections were immunohistochemically studied for CD34, S-100 protein, factor XIIIa, SMA (smooth muscle actin), and Ki67.

**Figure 1:**
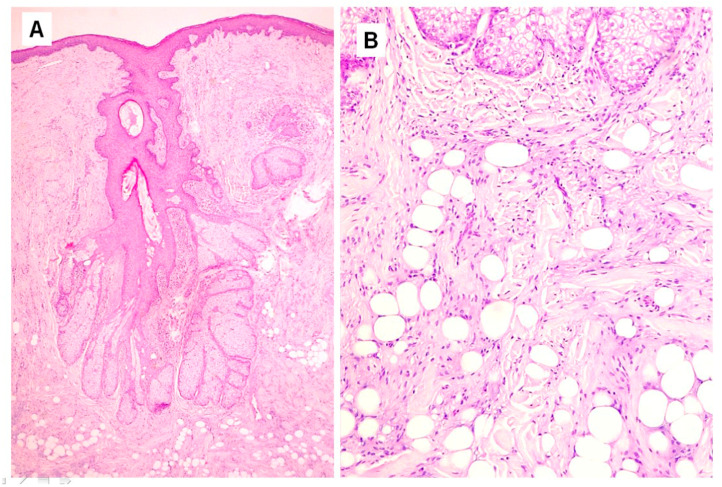
(**A**). A dilated follicular structure with irregular sebaceous glands (HandE, original magnification ×25) (**B**). Mesenchymal component with fibromyxoid stroma, spindle cells, collagen bundles, and mature adipocytes (HandE, original magnifications ×40).

**Figure 2:**
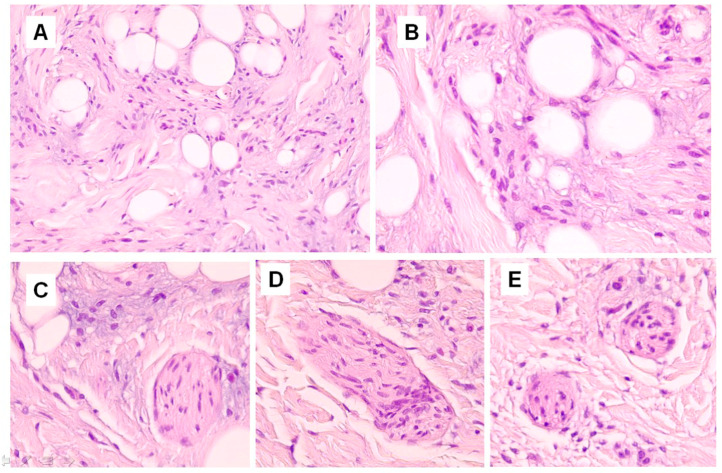
(**A**,**B**). Spindle cells, mature adipocytes, fibromyxoid stroma and collagen bands, mimicking spindle cell lipoma (HandE, original magnifications ×200, ×400). (**C**–**E**). Neural structures, mature adipocytes, spindle cells and fibrous stroma (HandE, original magnifications ×400).

**Figure 3:**
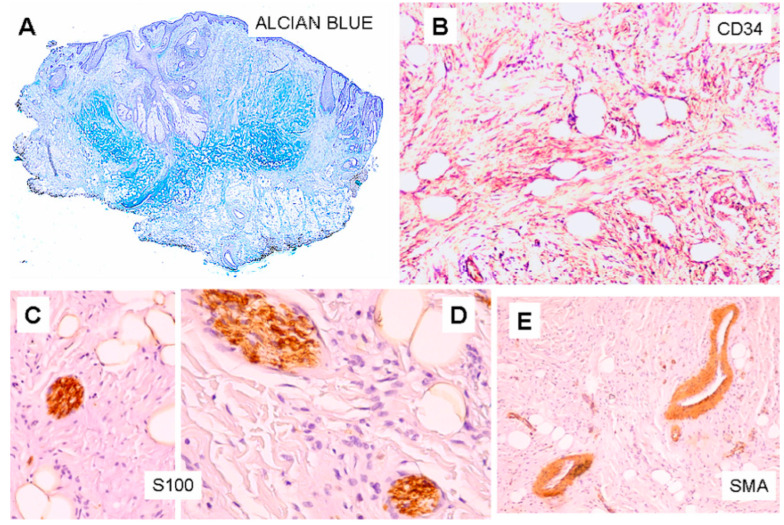
(**A**). Fibromyxoid stroma positive for Alcian blue (Alcian-blue stain, original magnification ×10). (**B**). Fibromyxoid stroma and spindle cells positive for CD34 (CD34 stain, original magnification ×200). (**C**,**D**). S100+ small nerve bundles and adipocytes (S100 stain, original magnification ×400). (**E**). Vascular structures positive for SMA (SMA stain, original magnification ×100).

Histopathological examination showed a dilated follicular structure, located in the dermis and extending to the upper portion of the subcutaneous fat; from this, multiple enlarged sebaceous lobules extended to the adjacent and underlying dermis (Figure 1A). This folliculosebaceous structure was connected to the overlying epidermis, that did not show relevant histologic alterations (Figure 1A). The degree of dilatation was mild–moderate, not configuring a true cyst. The central cavity was lined by a squamous epithelium, very similar to that of epidermis and of the infundibular segment of the hair follicle, showing basal, spinous, granular and horny layers (Figure 1A). The cavity appeared filled with laminar keratin, orthokeratotic and parakeratotic cornified cells, sebaceous material and some demodex. No structures referable to the pilar unit (lower segments of the follicle or piloerector muscles) or to eccrine or apocrine sweat glands were recognized. Radiating from the follicular structure, connected through short sebaceous ducts, there were a certain number of abnormal hypertrophic sebaceous glands (Figure 1A,B). They appeared as pyriform multilobulated lobules, composed of mature cells with a discontinuous single basal row of basaloid nonvacuolated sebocytes. The surrounding stroma was composed of mature dense fibrous tissue, containing small and medium vascular channels associated with a mild inflammatory infiltrate, consisting of lymphocytes with some plasmacells (Figure 1A). In the stroma, next to the follicular and sebaceous structures, there were islands of mature adipose tissue, represented by small aggregations of mature adipocytes (Figure 1A). In hematoxylin-eosin stained sections, short nerve bundles, embedded in the lesional stroma, were seen (Figure 2C–E). More deeply, the lesion displayed an Alcian blue-positive fibromyxoid stroma (Figure 3A), containing mature adipocytes and collagen bundles, mixed to spindle cells with single-elongated nuclei and bipolar cytoplasm, resembling spindle cell lipoma (Figure 1B and Figure 2A,B). Some foci of granulomatous inflammation were detected in the dermis. There were no evident clefts between the fibroepithelial structure and the surrounding altered stroma. Cytologic atypia and mitotic figures were not seen. Immunohistochemically, CD34 stained spindle cells and the fibromyxoid stroma (Figure 3B). The S100 protein stain revealed a greater number of neural structure than observable in the routine stained sections; they appeared as short neural bundles, variable in thickness, with diameters ranging from 0.05 and 0.20 mm, and disorderly distributed into the fibromixoid stroma, often unrelated to the vascular structures (Figure 3C,D). The S100 protein also positively stained mature adipocytes. Scattered spindle and dendritic stromal cells were positive for factor XIIIa; vascular structures, embedded in the mesenchymal component, showed immunoreactivity for SMA (Figure 3E); Ki67 positive spindle cells were less than 1%.

## 3. Discussion

Although lacking a characteristic clinical appearance, folliculosebaceous cystic hamartoma shows distinct histologic features. The basic parameters for the histopathologic diagnosis, originally stated by Kimura et al., were: (I) an infundibular cystic structure with connected sebaceous glands; (II) a compact fibroplasia around the epithelial component; (III) mesenchymal changes around the fibroepithelial structure, including fibrillary bundles of collagen, adipocytes, and an increased number of small venules; (IV) clefts between the fibroepithelial structure and the surrounding stroma and at the periphery between the altered stroma and the adjacent compressed fibrous tissue; (V) confinement of the process primarily to the dermis, but with a possible subcutaneous extension [1]. A high number of histopathological differential diagnoses are to be considered. They include dermoid cyst, steatocystoma (simplex), sebaceous hyperplasia, pilar sheath acanthoma, perifollicular fibroma/fibrous papule and fibrofolliculoma/trichodiscoma. A dermoid cyst may display a folliculosebaceous structure, but it is a true cyst, possibly associated with other adnexal structures (eccrine or apocrine glands), sometimes connected to the cystic space; the mesenchymal stroma, typical of folliculosebaceous cystic hamartoma, is lacking [6]. Steatocystoma shows a dermal cystic structure, lined by a corrugated lining, composed of stratified squamous epithelium, without a granular layer, with the characteristic presence of sebaceous glands included in or adjacent to the wall; a mesenchymal overgrowth is absent [8]. Sebaceous hyperplasia may resemble folliculosebaceous cystic hamartoma, because it shows a dilated follicular structure with associated sebaceous glands; however, these structures are more superficial and lack mesenchymal stroma [6]. Pilar sheath acanthoma appears as an infundibular dilatation, filled with abundant keratinous material and showing proliferating outer-root sheath epithelium, but not hypertrophic sebaceous glands [9]. Perifollicular fibroma/fibrous papule displays a fibrous stroma and dilated capillaries, sometimes rudimentary, distorted or dilated follicles filled with keratin, but sebaceous glands or lobules are absent [6]. Fibrofolliculoma and trichodiscoma, consisting of the same components, although in diverse arrangement and in different amounts, are considered to be stages of the same hamartomatous process, characterized by mantle differentiation (mantle-oma) [10]; fibrofolliculoma shows a dilated follicle filled with keratin, thin epithelial strands extending from the cystic structure, creating a fenestrated pattern, and fibrous stroma, composed of ribbon-like collagen bundles, mucin, and fibrocytes, but it lacks a relevant sebaceous component [6]. In trichodiscoma, the bulk of the lesion is a central stromal component, constituted by abnormal connective tissue, with characteristics similar to those of fibrofolliculoma, surrounded by clusters of distorted infundibula, epithelial cords and deformed sebaceous lobules [10]; a central infundibular cystic structure is absent.

Folliculosebaceous cystic hamartoma may also resemble trichofolliculoma and has been also considered a late stage of this benign neoplasm [11]; however, such a hypothesis has not been universally accepted, and these two lesions are mostly regarded as two distinct entities that should be differentiated [3]. Histopathologically, trichofolliculoma shows a dilated follicular structure with associated rudimentary follicles, but lacks a sebaceous component [6]. Moreover, sebaceous trichofolliculoma, considered a variant of trichofolliculoma, appears to show an even closer resemblance to folliculosebaceous cystic hamartoma because it displays a conspicuous sebaceous component [3]; sebaceous trichofolliculoma is characterized by a dilated follicular structure with rudimentary follicles associated with sebaceous glands, however, it is more superficially located; rudimentary follicles are connected with the cystic space, hair shafts are usually present within the follicular structures and a mesenchymal component is said to be absent [3,6]; these two lesions are very similar and have also been considered to represent a unique lesion [12].

The term “hamartoma”, derived from the Greek verb *hamartanein* (to fail, to err), refers to a malformation, consisting of abnormally arranged mature normal tissues, in an organ composed of identical tissues and cellular elements [13]. Therefore, cutaneous hamartomas are expected to show a variable admixture of epidermal, adnexal, dermal and hypodermal cells and tissues. Folliculosebaceous cystic hamartoma, a cutaneous hamartoma generally located in the dermis, is characterized by an infundibular cystic structure with numerous sebaceous lobules surrounded by a mesenchymal component, generally consisting of fibrous stroma, capillaries and small venules, malformed vascular channels, single or grouped adipocytes, and mature fat lobules [1,2,3,4,5,6,7].

In some lesions, showing the characteristic histologic epithelial features of folliculosebaceous cystic hamartoma, a conspicuous amount of mature fat tissue has been reported [14,15,16]. In such cases, large or very large clusters or confluent islands of adipocytes were observed among the dermal collagen fibers. Such complex lesions have been regarded as examples of a complex cutaneous hamartoma and considered rare associations of a *nevus lipomatosus superficialis* with a folliculosebaceous cystic hamartoma [14,15] or, alternatively, a *nevus lipomatosus superficialis* having a folliculosebaceous component [16]. Probably, these cases can be also interpreted as examples of folliculosebaceous cystic hamartoma having a very large adipocytic dermal component.

More rarely, folliculosebaceous cystic hamartoma also shows other elements, composed of other tissues and cells normally present in the skin, although arranged in abnormal structures. Such additional components include small superficial sebaceous lobules directly connected to the overlying epidermis, without associated pilar structures [6], immature fat cells with or without multiple lipid droplets (preadipocytes), observed near the sebaceous lobules [7], large areas of small, thick-walled, malformed vascular structures in tufted aggregates [9], dilated ducts with branching or tubular appearance, showing apocrine differentiation [9], cystically dilated hamartomatous apocrine glands [7], hair shaft fragments within the cystic structure [17], granulomatous inflammation caused by the rupture of the wall of infundibular structures [3], and abundant stromal mucin [3]. Moreover, a particular variant, labelled as folliculosebaceous cystic hamartoma with perifollicular mucinosis, showing large amounts of mucin, positively stained by colloidal iron and Alcian blue, was described in a 8-year-old boy [18]. In a unique case, reported under the term of folliculosebaceous smooth muscle hamartoma, the stromal component of the lesion appeared entirely composed of spindle cells, strongly positive for muscle-specific actin and for desmin, arranged in fascicles [19].

In the reported case, the epithelial component of the lesion was represented by an enlarged, dilated follicular structure with irregular sebaceous lobules. As expected, the mesenchymal component showed a dense fibrous stroma, numerous small and medium-caliber vascular structures and clusters of mature adipocytes. However, in addition to these classic features, in the stroma surrounding the pilosebaceous structure, a certain number of short neural bundles were seen. Moreover, in its deep portion, the lesion displayed a stromal mucinous Alcian blue-positive component, composed of normal adipocytes associated with spindle elongated cells with single elongated nuclei and collagen bundles, resembling spindle cell lipoma. As in some previous reported cases [5,18], clefts between the epithelial and the stromal components and between lesional and extralesional stroma were not seen. The immunohistochemical study showed some sparse dendritic and spindle stromal cells positive for factor XIIIa, according to previous reports [7]. The SMA stain showed no presence of smooth muscle tissue. The neural component was positive for the S100 protein stain and the spindle cell lipomatous component was intensely positive for CD34.

In folliculosebaceous cystic hamartoma, the occurrence of these two latter features seems to be rare. A neural component has been reported in only two previous cases. In 1993, Donati and Balus described a case of a 32-year-old woman with a pedunculated skin-colored nodule on the antitragus of her right ear, histologically showing small nerve branches [20]. In 1997, Toyoda and Morohashi described a female patient aged 55 with a dome-shaped nodule on her right cheek, histologically displaying a dense aggregation of thick trasversely-coursing nerve bundles in the deep portion [8]. In our case, the relatively small amount of neural tissue may raise the question whether the observed neural structures are only accidentally entrapped in the proliferating stroma or really represent an actual component of the lesion. However, the study of the published cases of folliculosebaceous hamartoma shows that the neural component, if present, is generally rather small. Donati and Balus, the first who reported such a feature, described it as “small nerve branches” (shown in their Figure 3), very similar to ours [20]. Toyoda and Morohashi reported a single aggregation of neural structures, consisting of neural bundles which were rather short, as shown in their Figure 5, that needed to be highlighted by asterisks [8]. Therefore, although of little amount, we considered the detected neural tissue as a real constituent of the lesion, because it was found embedded in the fibromixoid stromal component, did not always show a clear relationship with the vascular structures, did not form a regular neural network, and appeared to be haphazardly distributed in the fibrous tissue.

Spindle cell lipoma is a soft tissue benign tumor, described by Enzinger and Harvey in 1975. Histologically, the lesion may display various patterns. The classic form consists of a mixture of mature fat and spindle cells, showing elongated nuclei and cytoplasmic processes. These cells appear embedded in a mucoid matrix and are often mixed with characteristic birefringent collagen bundles [21].

A lipomatous component, showing a close resemblance to spindle cell lipoma, has been recently reported in two cases. In 2015, Nguyen, Skupsky and Cassarino reported a case of a 24-year-old man presenting a nasal papule, histologically showing a cystic follicular structure with multiple irregular sebaceous lobules, associated with a dermal mesenchymal component, consisting of mature adipocytes, fibromyxoid stroma, prominent collection of mucin, CD34+ spindle cells, and ropey collagen bundles, mimicking spindle cell lipoma [22]. More recently, Oulee and Cassarino described a male patient aged 36 with a lobulated nasal lesion, histologically displaying a large irregular folliculosebaceous structure, associated with a mesenchymal component, consisting of a proliferation of spindle-shaped cells, mucin and thickened, ropey-appearing collagen bundles, which are features resembling spindle cell lipoma [23].

In our case, the lipomatous spindle cell component appeared very similar to that observed in these case, although collagen bundles were shorter and thicker. Interestingly, a similar stromal component, composed of spindle cells, adipose tissue and prominent collagen bundles, resembling spindle cell lipoma, has been recently reported in cases of spindle cell predominant trichodiscomas [24], included in the trichodiscoma/fibrofolliculoma spectrum [24,25]. Such a peculiar mesenchymal component, shared by folliculosebaceous hamartoma and by fibrofolliculoma/trichodiscoma, can be interpreted as a specific type of spindle cell lipomatous metaplasia, occurring in different cutaneous hamartomas [24].

In conclusion, we reported a rare case of folliculosebaceous cystic hamartoma showing the association of a spindle cell lipomatous component with an neural component. To our knowledge, such an association has not been previously reported.

## Data Availability

The data presented in this study are available on request from the corresponding author.
